# MED23/404: The KASUS-Platform for Problem-Oriented Learning Groups: Using a digital camera and a Palm Pilot II to document clinical cases and share them over the WWW

**DOI:** 10.2196/jmir.1.suppl1.e65

**Published:** 1999-09-19

**Authors:** C Elsner, T Went, C Richter, H Kottkamp, G Hindricks

**Affiliations:** ^1^Heart Center LeipzigLeipzigGermany

**Keywords:** Problem-based Learning, Internet, Medical Education, Digital Camera, Palm Pilot, Kasus

## Abstract

**Introduction:**

In addition to regular seminars, the department for Internal Medicine at the Heart Center Leipzig uses Bedside-Teaching and Problem-Oriented Learning (POL) to teach medical students. The advantages of those teaching methods consist in their ability to foster active student inquiry and critical thinking. To have the chance to record interesting cases and reuse them for later seminars, the department beta-tested the PC/WWW-Platform KASUS. Acquiring a case and implementing it into the KASUS database for future use is achieved with a simple system consisting of a palmtop computer and a digital camera. Sharing the cases with other groups learning in the POL-format is possible with the KASUS ONLINE application and the WWW-based "Studentencafe", which allows students to share and discuss cases in different forums.

**Methods:**

The Platform (http://www.kasus.de) consists of two modules: One module is intended for case presentation and case-workup. A second module is intended for case material sharing, POL-group preceptor discussion and information exchange on POL. Both modules use static/dynamic HTML and can be used local or over a TCP/IP network. For the case acquirement a normal Palm Pilot II and a digital camera were used to document case histories and findings. The Palm Pilot II was equipped with a Jfile database which can be synchronized with the KASUS-Database.

**Results:**

Material for single cases could be acquired and the material could be presented in students´ seminars successfully. The Palm Pilot II performed good, but showed not to be fast enough to type in the case material. For digital case-documentation and the adequate case-authoring for POL-groups guidelines were put up. A small library with Acrobat Reader Files linked e.g. with recorded heart/lung sounds was collected for modular use with the system. The "Studentencafe" was launched in the internet. Existing POL groups at the University of Leipzig and the FU Berlin started using this module for information exchange and serve as content providers.

Discsussion:

With the combination of a palmtop computer and digital camera, case documentation and implementation in a case database is fast and effective. The advantages of the established platform lie in its utilization of the universal accessibility and network capabilities of the WWW. The use of an Internet web-browser interface provides platform-independent access and the opportunity to share and discuss case information from different remote locations. We believe that those applications can support POL groups in establishing intensive communication and can supplement their traditional POL learning approach by using the internet-support. Cases can be exchanged more easily, including complex multimedia case-related data. Facilitated information exchange on an inter-university level offers an opportunity to discuss with students coming from different backgrounds.

